# The de-implementation and persistence of low-value HIV prevention interventions in the United States: a cross-sectional study

**DOI:** 10.1186/s43058-020-00040-6

**Published:** 2020-06-30

**Authors:** Virginia R. McKay, Todd B. Combs, M. Margaret Dolcini, Ross C. Brownson

**Affiliations:** 1 Center for Public Health Systems Science, The Brown School, Washington University in St. Louis, St. Louis, MO USA; 2 Hallie E. Ford Center for Healthy Children and Families, College of Public Health and Human Sciences, Oregon State University, Corvallis, OR USA; 3 Prevention Research Center, The Brown School, Washington University in St. Louis, St. Louis, MO USA; 4 Department of Surgery (Division of Public Health Sciences) and Alvin J. Siteman Cancer Center, Washington University School of Medicine, Washington University in St. Louis, St. Louis, MO USA

**Keywords:** De-implementation, HIV prevention, Public health, Evidence-based intervention

## Abstract

**Background:**

As more effective or efficient interventions emerge out of scientific advancement to address a particular public health issue, it may be appropriate to de-implement low-value interventions, or interventions that are less effective or efficient. Furthermore, factors that contribute to appropriate de-implementation are not well identified. We examined the extent to which low-value interventions were de-implemented among public health organizations providing HIV prevention services, as well as explored socio-economic, organizational, and intervention characteristics associated with de-implementation.

**Methods:**

We conducted an online cross-sectional survey from the fall of 2017 to the spring of 2019 with organizations (*N* = 188) providing HIV prevention services in the USA. Organizations were recruited from the Center for Disease Control and Prevention’s (CDC) website gettested.org from 20 metropolitan statistical areas with the highest HIV incidence. An organization was eligible to participate if the organization had provided at least one of the HIV prevention interventions identified as inefficient by the CDC in the last ten years, and one administrator familiar with HIV prevention programming at the organization was recruited to respond. Complete responses were analyzed to describe intervention de-implementation and identify organizational and intervention characteristics associated with de-implementation using logistic regression.

**Results:**

Organizations reported 359 instances of implementing low-value interventions. Out of the low-value interventions implemented, approximately 57% were group, 34% were individual, and 5% were community interventions. Of interventions implemented, 46% had been de-implemented. Although we examined a number of intervention and organizational factors thought to be associated with de-implementation, the only factor statistically associated with de-implementation was organization size, with larger organizations—those with 50+ FTEs—being 3.1 times more likely to de-implement than smaller organizations (95% CI 1.3–7.5).

**Conclusions:**

While low-value interventions are frequently de-implemented among HIV prevention organizations, many persisted representing substantial inefficiency in HIV prevention service delivery. Further exploration is needed to understand why organizations may opt to continue low-value interventions and the factors that lead to de-implementation.

Contributions to the literature

- This study demonstrated persistence of low-value interventions in HIV prevention was common among HIV service organizations.
- The findings of this paper also revealed that, with the exception of organization size, there are no significant unifying factors that separate organizations that de-implement low-value interventions from those who do not.
- Finally, our findings suggest that active approaches will likely be needed to encourage appropriate de-implementation rather than assuming de-implementation will occur through passive knowledge dissemination.

## Background

De-implementation is the process by which interventions and programs are discontinued and is an emerging line of inquiry within dissemination and implementation (D&I) research [1, 2]. Existing evidence suggests that de-implementation occurs in public health systems, but the extent to which de-implementation occurs when appropriate remains unclear [2–4]. Ideally, low-value interventions are de-implemented and replaced when either more effective interventions are available or more efficient, that is more cost-effective or streamlined, interventions are available, but well-integrated, low-value interventions persist in practice [1, 5]. Although there is considerable evidence that a multitude of factors at socio-economic, organizational, practitioner, and intervention levels contribute to the adoption and implementation of interventions [6], evidence outlining factors that make de-implementation more likely is limited in public health settings. Organizational capacity to provide services, for instance, funding, staff, and adequate collaborations with external partners are the most proximal factors contributing to service delivery [7]. In addition, characteristics of interventions, such as interventions that are complicated or burdensome to provide may make de-implementation more or less likely [1].

HIV prevention provides an ideal public health context for exploring de-implementation of low-value interventions. Investment in HIV prevention research has led to several waves of evidence-based interventions over the last three decades [8–11]. The Centers for Disease Control and Prevention (CDC) has spearheaded HIV prevention efforts in the US by identifying the most effective and efficient interventions using rigorous effectiveness criteria, and then disseminating identified interventions [9]. Historically, intervention has been disseminated by the CDC through two key policies. The first policy, the Diffusion of Effective Behavioral Interventions for HIV prevention (DEBI), beginning in 2002, supported an array of over 30 interventions aimed primarily at reducing sexual risk behaviors available for individual, group, and community levels of intervention as well as targeting different populations at increased risk for HIV in the US like intravenous drug users, specific ethnic and racial minorities, and men who have sex with men [12, 13], and more interventions were added in subsequent years. Drawing on the best available evidence at the time, uptake of interventions included as part of the DEBI policy was substantial and made evidence-based intervention ubiquitous in HIV prevention practice. These interventions have been disseminated to approximately 11,300 organizations including public health departments, community-based organizations, and medical clinics over the last 15 years [10]. In supporting the DEBI policy, the CDC has invested approximately $100 million dollars identifying and disseminating HIV prevention interventions per year [10].

In 2012, the CDC revised HIV prevention intervention recommendations as part of the High Impact Prevention (HIP) policy. With emerging evidence supporting the effectiveness of biomedical interventions, especially pre-exposure prophylaxis, and evidence lacking for the effectiveness of some behavioral interventions, the HIP policy focused more heavily on expanding HIV testing and engaging/retaining HIV-positive individuals in clinical care [14]. While some interventions recommended as part of the DEBI policy are still considered the most effective and were subsumed under HIP, approximately 37 interventions are considered low value, meaning they are not the most effective or efficient to provide and are no longer recommended [15]. Although public health organizations were not overtly encouraged to de-implement low-value interventions no longer recommended, financial support (a key factor leading to sustainment and de-implementation) was redirected toward HIP interventions [7, 16]. However, it is unclear if low-value HIV prevention interventions persist in public health organizations.

With the goal of quantitatively characterizing the extent of de-implementation through a broad survey of public health organizations involved in HIV prevention, we examined the extent to which low-value interventions were de-implemented among public health organizations providing HIV prevention services, as well as explored socio-economic, organizational, and intervention characteristics associated with de-implementation.

## Methods

We report data from the quantitative first stage of a sequential mixed-methods study. We conducted a cross-sectional survey with 188 organizations through Qualtrics [17]. Organizations were identified through the CDC’s website gettested.cdc.gov, a searchable website where organizations can register as providing an HIV test, a core HIV prevention and treatment service [18]. We searched for organizations within a 50-mile radius, the widest search radius available, from the 20 metropolitan statistical areas (MSAs) with the highest HIV incidence. These 20 areas encompass approximately 50% of all new HIV diagnoses in the US [19]. All research protocols were reviewed and approved by the Institutional Review Board at Washington University in St. Louis. When required, we also received approval from individual states to conduct research with local health departments. We used the STROBE and CHERRIES checklists for cross-sectional and online studies to develop and report our results in the current manuscript [20, 21].

### Recruitment and eligibility

**Fig. 1:**
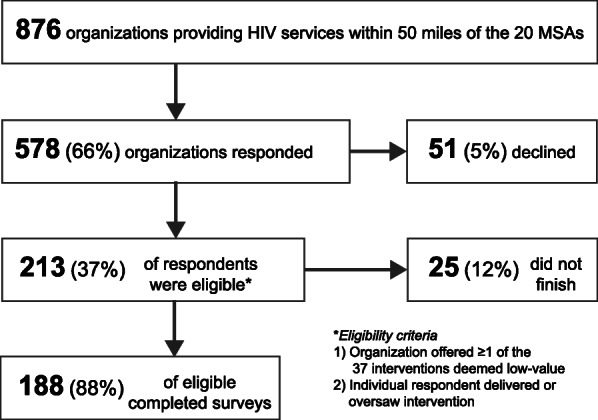
Response, eligibility, and completion rates

All organizations were recruited for participation continuously over the data collection period. Organizations were eligible if one of 37 low-value interventions defined as low value by the CDC had been implemented. Interventions included as part of the eligibility criteria are listed in Appendix A. Those reported as implemented are also shown in Fig. 1. Executive directors or supervisors who oversee HIV prevention services were recruited to complete the survey either by phone or email. If organizations had multiple locations, one person from the organization was asked to respond on behalf of all locations. Participants agreeing to participate were given a specific survey link. The consent document was provided at the start of the survey and in the recruitment email describing the purpose of the survey, the principal investigators, the length of the study, time allowed to respond, and details of data storage and reporting. Participants were asked an initial set of screening questions as part of the survey to determine (1) if the agency implemented a low-value intervention within the last ten years, and (2) if the contact was the most appropriate person to complete the full survey. If the recruited person indicated that they were not the most appropriate person to respond to the survey, we contacted the participant again and asked to be referred to the most appropriate staff member. Staff members were contacted a maximum of three times before considered a non-respondent.

### Survey design and data collection

All data were self-reported via a survey conducted online through Qualtrics. Items for the survey were generated from existing questions [6] and existing implementation frameworks. Three frameworks were used: Meyer, Davis, and Mays’ conceptual framework for Organizational Capacity [7], Scheirer and Dearing’s framework for Sustainability of Public Health Programs [22], and Fixsen and colleagues’ implementation framework [4, 23]. Synthesized together, these frameworks identify the predictors of de-implementation within organizations, de-implementation of interventions as an outcome, and the downstream influences of de-implementation on communities and organizations as well as the processes involved in the de-implementation. Meyer and colleagues' Organizational Capacity Framework identifies central concepts (e.g., financial, human, and physical resources) indicated by specific variables essential to implementing and sustaining interventions over time. For example, financial resources are indicated by both level and source (external vs. internal). A reduction in capacity in one or more areas may lead an organization to de-implement an intervention [7]. Our previous work and the Sustainability framework (the converse of de-implementation) suggests that when EBIs are de-implemented, there are subsequent outcomes for organizations, staff, clients and communities [1, 22]. The Implementation Framework captures the detailed processes involved with implementing and sustaining interventions in organizations [23]. We extended this model to incorporate a de-implementation phase that describes the process of de-implementation and a set of activities likely to take place when de-implementation is occurring [4]. Survey questions are available in Appendix B.

The survey protocol was piloted in a separate set of locales with approximately 20 executive directors and supervisors of HIV prevention services to identify any problems with the survey structure and flow. The survey question format was divided into several subsections: characteristics of the organization (e.g., type, size, client population) and of the respondent (e.g., age, gender, and race/ethnicity) (15 items), details of initial intervention implementation, and whether the intervention was continued or de-implemented (2 items). Then, the respondent was asked a series of questions for each intervention reported as continued (3 items) and each intervention reported as de-implemented (10 items). Depending on the number of interventions on which the participant was reporting, the total number of items ranged from 30 to 150 items. Surveys took on average 20 min to complete, ranging 15–30 min. To reduce reporting bias, participants were made aware that their responses would be kept confidential from their organization and when reporting study results. Participants were given a $20 gift card as incentive for completing the survey. Participants were able to change their answers through a back button. Participants were reminded up to three times to complete the survey, and the survey was closed if not completed after three weeks from the final reminder. Data were collected from the fall of 2017 to the spring of 2019.

### Measures

Our primary goal was to describe the continuation or de-implementation of low-value interventions that had been adopted by HIV services organizations over the previous 10-year period. We looked only at the 37 interventions deemed low-value by the CDC, and examined the likelihood of intervention de-implementation based on organizational characteristics specifically organizational type, size, collaboration partners, funding sources, and type of intervention. Respondents chose their organization type in response to the question, “How would you best describe your organization?” with available choices of a community-based organization (CBO), Clinic, public health department (PHD), or other. Ten organizations chose “Other” and these were recoded into the appropriate categories. Most were either “non-profit” or some type of “hospital”, reclassified as CBO or Clinic. As an indication of organizational size, we used full-time equivalent (FTE). FTE was reported as a continuous variable and was collapsed into a range of categories (0–10, 11–50, 50 or more). We chose to collapse this variable as half of the organizations had fewer than 50 FTEs, three-quarters had fewer than 100, and two organizations were disproportionately large (10,000 and 34,000 FTEs). Our primary concern was whether small or large organizations would be more likely to de-implement interventions, rather than the specific number of FTEs. Respondents were asked if collaborations were either formal or informal with a number of other organizational types (a college or university, other health service agency, social service agency, faith-based organization, for-profit business, local or state health department, or other) and funding sources (federal government, state government, non-profit, community/individual donations, insurance billing, direct fees, or other). An intervention was categorized as individual-level if the interventions was intended to be delivered one-on-one with a client, a group-level if the intervention was intended to be delivered with more than one client, and a community-level if the intervention was intended to be delivered with a number of people outside of the organization.

### Statistical analysis

We downloaded data from Qualtrics and used the R statistical environment for analysis [24]. Complete responses were used for analyses. We provide descriptive statistics indicating the frequency, type of interventions implemented, and whether they were continued or de-implemented. Organizational and intervention characteristics and their association with de-implementation were investigated via logistic regression.

## Results

### Organizations and participants

We identified 876 HIV service providers within 50 miles of the 20 metropolitan areas, recruited all of them via phone or email or both, and achieved a response rate of 66% (*N* = 578), see Fig. 1. Of organizations that responded, 38% (*N* = 213) met the eligibility criteria, meaning they implemented one of the low-value interventions we identified. Five percent (*N* = 51) of organizations declined to participate. Of the 213 eligible respondents, 12% (*N* = 25) did not finish the survey, leaving 188 eligible responses available for analysis. Respondents representing organizations were diverse in race and ethnicity (25% black, 34% white, 22% Hispanic, and 8% other), education level (15% < 4-year degree, 27% 4-year degree, 59% graduate degree), and age (38% 20–39 years, 47% 40–59 years, 15% 60+ years). Participants were 62% female and reported an average of 11.1 years with their current agency.

Table 1 shows the interventions reported by intervention level, organization type, and organization size. A slight majority of organizations were community-based (52.7%), while 29.8% were clinics, including hospitals and federally qualified healthcare centers, and the remaining 17.6% were state or local departments of health. About one fifth (21%) of the organizations had fewer than 10 FTEs, 30% had 10 to 50 FTEs, and the remaining 41% had more than 50 FTEs. Most organizations had active collaborations of one kind or another, most frequently social (80.9%) or health service (88.8%) organizations, and 63.3% of organizations had collaborations with a college or university. Most reported a variety of funding sources, with state or local (88.3%) and federal (73.9%) funds being most often reported. While there was much variation in the percentage of priority populations served, low socio-economic status and racial or ethnic minority individuals on average comprised the largest portions of clientele.

**Table 1:** Characteristics of 188 eligible respondent organizations

Organization type	Total (%)
Community-based organization	99 (52.7)
Clinic	56 (29.8)
Department of Public Health	33 (17.6)
**Organization size (FTEs)**	
1–10	41 (21.4)
10–50	57 (30.3)
50+	77 (41.2)
Not reported	13 (5.0)
**Organization collaborations**	
Colleges or universities	119 (63.3)
Health service organizations	167 (88.8)
Social service organizations	152 (80.9)
**Funding sources**	
Federal channels	139 (73.9)
State or local channels	166 (88.3)
Non-profit organizations	109 (58.0)
Donations from community	104 (55.3)
**Populations served (% clients)**	**Mean (SD)**
Low socio-economic status	64.6 (34.1)
Racial or ethnic minorities	64.6 (29.9)
Youth and young adults (< 30)	37.9 (27.4)
Men who have sex with men	35.9 (28.1)
HIV-positive individuals	34.5 (35.7)
Injection drug users	13.0 (17.7)
Transgender individuals	6.75 (9.32)

Notes: Percentages may not sum to 100 due to rounding. *FTE* full-time equivalent staff, *SD* standard deviation

### Low-value interventions implemented

Over the previous 10 years, organizations adopted a variety of interventions initially recommended but subsequently deemed low value. In all, the 188 organizations reported initially implementing 359 low-value interventions, most of which were group-level (56.8%), and the smallest number 16 (4.5%) were community-level interventions (Table 2). About half of the organizations (51%; *N* = 95) offered only one of the interventions, and the remaining 93 offered two or more. The average number of interventions implemented per organization was 1.91 (SD = 1.4), the median number of interventions was 1.

**Table 2:** Low-value interventions reported by level and by organization type and size

	Interventions	Intervention level		
		Group	Individual	Community
**Organization type**				
Community-based organization	179 (49.9)	103 (28.7)	70 (19.5)	6 (1.7)
Clinic	119 (33.1)	67 (18.7)	45 (12.5)	7 (1.9)
Department of Public Health	61 (17.0)	34 (9.5)	24 (6.7)	3 (1.0)
**Organization size (FTEs)**				
1–10	77 (21.4)	47 (13.1)	28 (7.8)	2 (1.0)
10–50	116 (32.3)	67 (18.7)	45 (12.5)	4 (1.1)
50+	148 (41.2)	81 (22.6)	57 (15.9)	10 (2.8)
Not reported	18 (5.0)	9 (2.5)	9 (2.5)	0
**Totals**	**359 (100)**	**204 (56.8)**	**139 (38.7)**	**16 (4.5)**

Notes: Percentages in parentheses may not sum to 100 due to rounding. *FTE* full-time equivalent staff. Percentages for intervention types by each of organization type and FTEs (group, individual, community) = percentage of ALL interventions

**Fig. 2:**
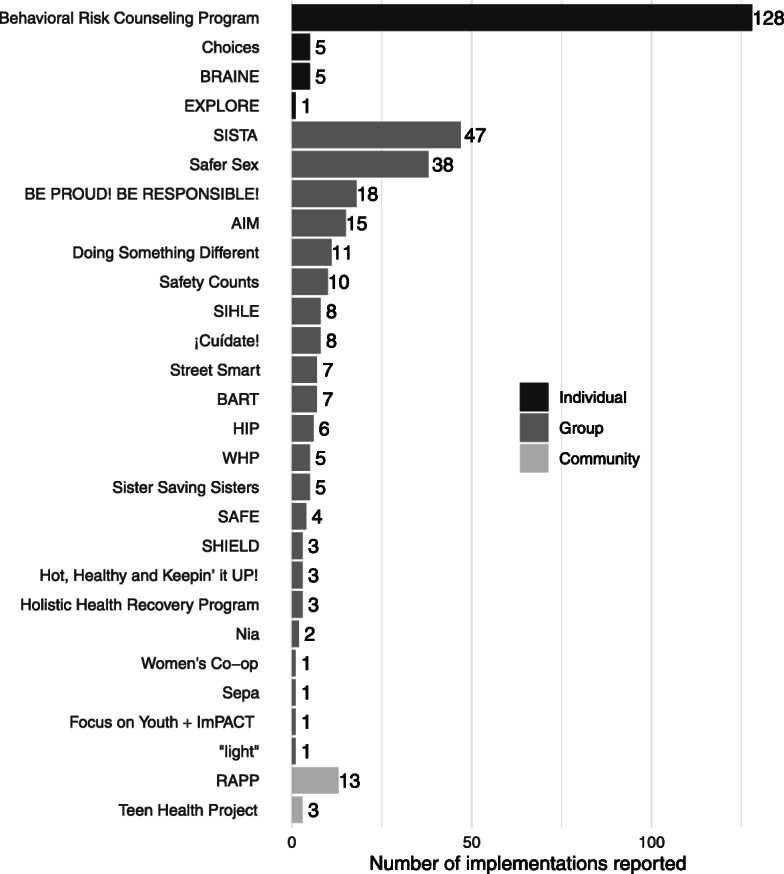
Implementation of low-value interventions, by intervention

Figure 2 shows the number of times each specific intervention was adopted and implemented by the responding organizations. The most frequently reported intervention was Behavioral Risk Counseling, an individual-level intervention reported by 128 agencies (68%); however, a variety of group-level interventions were implemented (22 different interventions). Of these, SISTA, an intervention designed for black women, and Safer Sex, an intervention designed for youth, were reported most often, by 47 (25%) and 38 (38%) organizations, respectively.

**Fig. 3:**
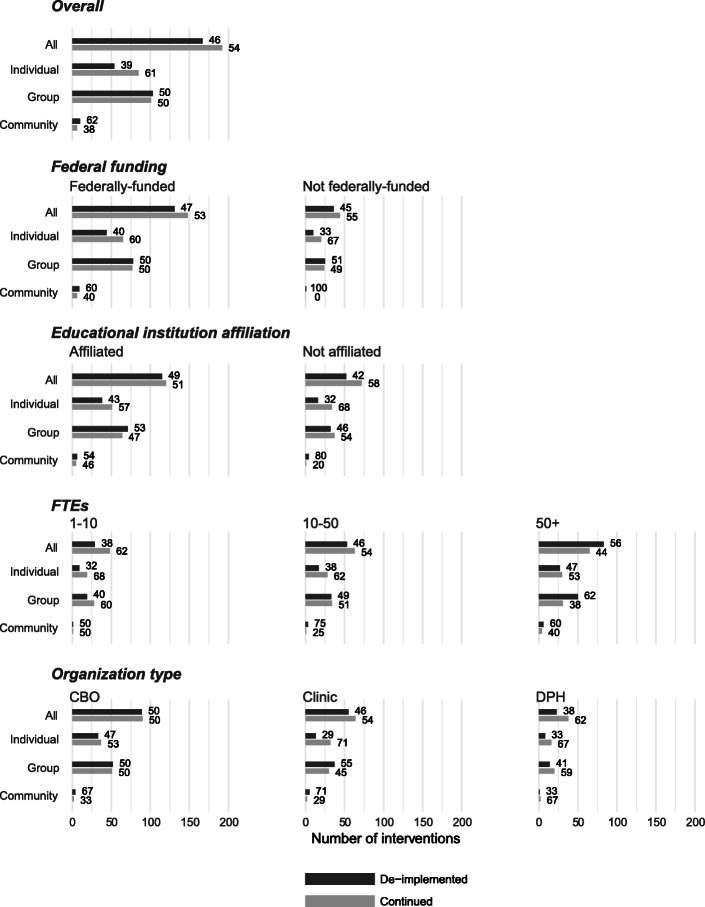
Implementation status of low-value interventions by organizational characteristics and intervention type. Bars represent the number of interventions, and numbers next to bars represent the percentage at each intervention level

Figure 3 shows the current implementation status—de-implemented or continued—of reported interventions overall and by characteristics of the organizations. The first row of the bar chart shows that overall almost half of the 359 low-value interventions were de-implemented (46%) indicated by the dark bars and 54% continued. By intervention type, 39% of individual interventions were de-implemented, while half (50% or 102) of group-level and 10 of the 16 (62%) reported community-level interventions were de-implemented. Below that examining federal funding support and academic affiliation, we observed very little variation in intervention de-implementation between organizations that received federal funding or had an academic affiliation and those that did not. The fourth row of charts examining organizational size shows that smaller organizations, those with 1–10 or 10–50 FTEs, de-implemented at smaller rates than their larger counterparts: 38% of interventions were de-implemented in organizations with 10 or fewer FTEs. In the bottom row of Fig. 3, we see that CBOs had the highest rates of de-implementation (50%) and departments of public health had the lowest rates (38%). Clinics, with similar numbers of group and individual interventions, had almost double the rate of de-implementation for group-level programs (55%) than that for individual ones (29%).

To examine the impact of these factors on de-implementation, we conducted a logistic regression (Table 3).

**Table 3:** Logistic regression results: outcome = de-implementation of low-value interventions

Effect	Odds ratio	95% CI
Community-based organization	Reference	
Clinic	0.6	[0.3–1.1]
Department of Public Health	0.6	[0.2–1.4]
FTEs, 1–10	Reference	
FTEs, 10–50	1.8	[0.8–4.4]
**FTEs, 50+**	**3.1**	**[1.3–7.4]**
Receives federal funds	0.6	[0.3–1.4]
Affiliated with college or university		[0.8–2.9]
Intervention level: individual	Reference	
Intervention level: group	1.5	[0.9–2.4]
Intervention level: community	2.0	[0.6–6.5]
Number of interventions reported	1.2	[0.9–1.5]
Log-likelihood (df = 10)	− 224.3	
AIC	468.5	
A.1.1.1.1.1.1. *N*	341	

Note: Standard errors used to create confidence intervals are clustered within organizations

The outcome for “success” was de-implementation of a low-value intervention. We included organizational characteristics—organization type, size in FTEs, and binary variables for whether federal US funding was received and the existence of an affiliation with a college or university—along with the intervention level and the total number of interventions reported by each organization. Standard errors are clustered within organizations to account for any association among the interventions offered at a particular organization and their likelihood of de-implementation. Of the 93 organizations that offered two or more interventions, 59 organizations reported all of those offered had the same status (either all continued or all de-implemented) and the remaining 34 organizations reported different statuses for interventions. From the table, we see that all else equal, organization type, whether the organization was federally funded or affiliated with an educational institution, the level of the intervention, or the number of interventions offered was not associated with the probability of de-implementation at a 95% confidence level. However, organization size was related to the likelihood of ending programs. Controlling for the other factors, organizations with 50 or more FTEs were about three times as likely as small organizations (those with 10 or fewer FTEs) to de-implement programs.

## Discussion

Understanding the de-implementation of low-value interventions is essential as we continue to invest in basic research and promote the use of effective interventions in public health practice. There are still only a handful of studies examining de-implementation in the context of public health [4], and we extend this work by describing the extent to which de-implementation occurs for a public health issue with a wide array of low-value interventions (i.e., HIV) and describe factors that may (or may not) be associated with de-implementation.

Our results demonstrate that a range of organizations serving a variety of populations implemented a number of interventions, which were the best available interventions at the time. Clinics and CBOs were more likely to have implemented these interventions, likely because large municipal public health departments often give small grants to provide services rather than provide services directly. Organizations opted to continue these low-value interventions, now no longer considered the most effective or efficient, approximately half of the time. This rate of low-value intervention persistence represents substantial inefficient use of an organization’s capacity to provide services both in terms of staff time and money spent. We found this rate surprising since evidence against these interventions and subsequent loss of CDC support for these interventions occurred beginning in approximately 2014 [10].

Understanding the potential drivers of de-implementation in addition to the scientific evidence is an important precursor to successfully stimulating appropriate de-implementation [25]. Toward this end, we examined whether specific organizational characteristics predicted appropriate de-implementation; however, very few organizational characteristics were associated with de-implementing low-value interventions. Organizational size was the only significant predictor of appropriate de-implementation with larger organizations (50 or more FTEs) more likely to de-implement low-value interventions. Larger organizations may have greater general capacity to make service delivery changes more quickly or have larger information networks, which leads to faster dissemination of information and evidence [7, 26].

Of equal interest is the number of organizational factors that did not demonstrate an association with de-implementation, namely academic partnerships or receipt of federal US funding. Ideally, academic partners and national agencies would support the dissemination of the most current evidence for or against any given intervention and incentivize change through funding mechanisms and technical support. This suggests external dissemination efforts to raise awareness about low-value interventions and availability of more efficient or effective interventions is inadequate or insufficient in promoting change similar to adoption of interventions. Furthermore, withdrawal of support by a national-level government agency, like the CDC, even after several years for specific interventions, is also likely insufficient to promote de-implementation among a substantial number of organizations. Organizations like clinics and CBOs, which have multiple funding streams, may opt to continue supporting an intervention through other means if one particular source of funding ends. Lastly, while descriptive statistics initially suggested that individual-level interventions were more likely to be continued and group- and community-level interventions de-implemented, this influence disappeared after controlling for organizational factors, namely organizational size. However, we do not consider these results definitive and would encourage others to continue exploring these factors to either confirm or refute our results in different public health settings with different types of interventions. Qualitative methods in particular with rich data collection could potentially help elucidate and explain relationships observed in this study.

Our study highlights a need to develop strategies that actively encourage appropriate de-implementation of ineffective or inefficient interventions. Healthcare settings, where research focusing on de-implementation and reduction of excess healthcare interventions has been ongoing for some time may provide promising approaches to de-implementation that could be tested in public [27]. As others have noted, the process for selecting which interventions to provide or end is nuanced, and administrators and staff may be considering multiple factors given the perceived needs of the community, the multitude of intervention possibilities, and the capacity of the organization to make service delivery changes [28]. Understanding from a staff perspective the reasons for continuing or de-implementing interventions and how staff make decisions to choose certain services over others is critical. Furthermore, understanding from a staff perspective why these interventions may continue to hold value will be helpful for public health scientists to develop useful strategies for encouraging de-implementation and support public health practitioners in their effort to provide the most effective services in the most efficient manner possible [7].

### Limitations

There were several limitations to the study. The study was not a random sample of all organizations delivering HIV services, which reduces the generalizability of the results. However, our wide coverage of areas nationally with high HIV incidence and diverse set of organizations helps ensure that we captured an appropriate cross section of organizations involved in HIV prevention. The study was conducted in a single country which limits the generalizability to other countries. Responses were self-report, which may have led to some recall bias. Also, while funding was often cited as a reason to end or continue interventions, we did not have data on funding amounts by source, which would have added to our analyses and findings.

## Conclusion

In the emerging field of de-implementation in public health, we demonstrated that while appropriate de-implementation of low-value interventions occurs, intervention persistence also occurs at a high rate. We also demonstrated that few organizational characteristics predict appropriate intervention de-implementation. Identification of other factors driving service delivery decisions is needed to improve the appropriate de-implementation of low-value interventions, enhance the uptake of more effective programs, and ensure more cost-effective investment in public health.

## Supplementary information

**Additional file 1:.** Appendix A HIV prevention interventions considered low-value due to lack of efficiency or evidence. Appendix B. Survey instrument

## Data Availability

Data is available upon reasonable request to the first author.

## Abbreviations

CBO: Community-based organization
CDC: Centers for Disease Control and Prevention
DEBI: Diffusion of Effective Behavioral Interventions
D&I: Dissemination and implementation
FTE: Full-time equivalent
HIP: High Impact Prevention
HIV: Human immunodeficiency virus
PHD: Public health department
MSA: Metropolitan statistical area
