# Supplementary material for: The de-implementation and persistence of low-value HIV prevention interventions in the United States: a cross-sectional study
Source: Implement Sci Commun. 2020 Jun 30;1:60. doi: 10.1186/s43058-020-00040-6 (PMC7427853; doi:10.1186/s43058-020-00040-6)
Supplement: Supplementary file 1 — Additional file 1:. Appendix A HIV prevention interventions considered low-value due to lack of efficiency or evidence. Appendix B. Survey instrument [file 43058_2020_40_MOESM1_ESM.docx]

**Appendix A**HIV prevention interventions considered low-value due to lack of efficiency or evidence

|  | **Intervention** | **Level** | **Brief Description** |
| --- | --- | --- | --- |
| 1 | RESPECT | Individual | RESPECT is an individual-level, client-focused, HIV prevention intervention, consisting of two brief interactive counseling sessions. |
| 2 | Doing Something Different | Group | Doing Something Different consists of one single skill-building group session to encourage change in norms, expectations, and social skills for promoting safer sex and condom use. |
| 3 | "light" | Group | Living in Good Health Together or “light” is a 7-session, small-group HIV-risk reduction intervention to stimulate motivation for behavior change along with individualized skill building required to accomplish personal HIV-related goals. |
| 4 | AIM | Group | Project AIM is a group-level youth development intervention designed to reduce HIV risk behaviors among youth. AIM encourages at-risk youth to imagine a positive future and discuss how current risk behaviors can be a barrier to a successful adulthood. |
| 5 | ARK | Group | Assisting in Rehabilitating Kids (ARK) is a small group, 12-session intervention consisting of educational, behavioral skills training, and motivational risk-sensitization manipulation components designed to reduce and maintain reductions of risky sexual behaviors among substance-dependent adolescents. |
| 6 | BART | Group | Becoming a Responsible Teen (BART) is a group-level, education and behavior skills training intervention designed to reduce risky sexual behaviors and improve safer sex skills among African American adolescents. In addition, the intervention encourages participants to share the information they learn with their friends and family and to provide support for their peers to reduce risky behaviors. |
| 7 | BE PROUD! BE RESPONSIBLE! | Group | Be Proud! Be Responsible! is a small group skills building and motivational intervention to increase knowledge of AIDS and sexually transmitted diseases (STDs) and to reduce positive attitudes and intentions toward risky sexual behaviors among African-American male adolescents. |
| 8 | cuidate | Group | ¡Cuídate! consists of six 1-hour modules delivered over a minimum of 2 days to groups of 6 to 10 youth. ¡Cuídate! can be delivered in community centers, schools, etc. by health educators, counselors, health care providers, etc. HIV/AIDS knowledge, condom negotiation, refusal of sex, and correct condom use skills are taught through interactive games, group discussion, role-plays, video, music, and mini-lectures. |
| 9 | FOY | Group | Focus on Youth (FOY) is an 8-session intervention delivered to small naturally formed peer friendship groups (3-10 youths) via discussions, games, and multimedia formats. conducted. The intervention is followed by monthly and annual booster sessions in which youth are given specific challenges to work through to reinforce the skills (e.g., decision making, communication, and condom use) they acquire in the primary sessions. |
| 10 | FOY+ImPACT | Group | Focus on Youth (FOY) is a community-based, eight session group intervention that provides youth with the skills and knowledge they need to protect themselves from HIV and other STDs. The curriculum, founded on the Protection Motivation Theory, uses fun, interactive activities such as games, role plays and discussions to convey prevention knowledge and skills. FOY targets African American youth, ages 12-15. |
| 11 | SIHLE | Group | SIHLE is a peer-led, social-skills training intervention aimed at reducing HIV sexual risk behavior among sexually active, African American teenage females, ages 14-18. An adaptation of the SISTA intervention, SIHLE emphasizes ethnic and gender pride, and enhances awareness of HIV risk reduction strategies such as abstaining from sex, using condoms consistently, and having fewer sex partners. |
| 12 | Street Smart | Group | A multi-session, skills-building program to help youth practice safer sexual behaviors and reduce substance use. Sessions address improving youths' social skills, assertiveness and coping through exercises on problem solving, identifying triggers, and reducing harmful behaviors. Agency staff also provide individual counseling and trips to community health providers. |
| 13 | Teen Health Project | Community | The Teen Health Project (THP) is a community-level HIV-prevention intervention that helps adolescents develop skills to enact change in themselves and the community. The program provides continued modeling, peer norm, and social reinforcement for the prevention of HIV risk behavior. The parents of participants are offered an educational workshop focused on HIV/AIDS information and approaches to discussing abstinence and condom use with their children. |
| 14 | TLC | Group | TLC (Together Learning Choices, previously referred to as Teens Linked to Care) is a small- group intervention designed for youth and young adults living with HIV. HIV-positive youth identify their risk behavior triggers and modify their patterns of substance use as well as increase self-efficacy of condom use and negotiation skills. |
| 15 | CE-AP | Group | The Communal Effectance—AIDS Prevention (CE-AP) intervention is a small group (3–6 women) intervention that emphasizes negotiation skills training and the idea that women’s sexual behavior not only affects themselves but also those around them. Women are taught to protect themselves from HIV infection through cognitive rehearsals, role plays, discussions, and interactive videos. |
| 16 | Choices | Individual | Positive Choice is an individual-level, interactive computer-based intervention to improve screening and counseling about ongoing sex risk and substance use among HIV-positive patients. |
| 17 | FIO | Group | FIO (Future Is Ours) is a small group, cognitive-behavioral intervention.  The intervention provides women with the skills necessary to communicate and negotiate safer sex with their partners (including how to identify and respond to abuse in relationships), and how to solve problems to avoid relapses. |
| 18 | Insights | Individual | Insights is an individually-tailored minimal self-help intervention that consists of two prevention packets mailed to participants three months apart. The information in the packets is tailored to the individual based on a baseline risk assessment. The magazine-style booklet includes non-tailored and tailored elements. The tailored elements are pulled from a “library” of all possible prevention messages to coordinate with responses from the baseline risk assessment survey. |
| 19 | SAFE | Group | The Standard SAFE intervention is a small group, motivational and skill building intervention to reduce risky sexual behaviors and STDs among minority women. The 3 intervention sessions, delivered to groups of 5–6 women, emphasize recognizing risk, increasing commitment to change behavior, and facilitating the acquisition of protective skills. Standard STD counseling and testing is also provided to everyone by a nurse clinician. |
| 20 | Safer Sex | Group | Safer Sex is an individualized skills-building intervention designed to increase condom use, reduce other risky sexual behaviors and prevent recurrent STDs among female adolescents. |
| 21 | Sepa | Group | SEPA is a six-session, culturally-tailored, small-group, skills building intervention designed to prevent high-risk sexual behaviors among low-income Mexican and Puerto Rican women. The intervention, delivered to groups of 11-13 women, promotes self-efficacy, builds skills and focuses on topics including: HIV/AIDS in the community, human anatomy and sexuality, education about HIV and other STDs, condom use, negotiation of safer sex, and preventing domestic violence. |
| 22 | SISTA | Group | This group-level, gender- and culturally- relevant intervention, is designed to increase condom use with African American women. Five peer-led group sessions are conducted that focus on ethnic and gender pride, HIV knowledge, and skills training around sexual risk reduction behaviors and decision making. |
| 23 | Sister Saving Sisters | Group | The Sisters Saving Sisters intervention is a single-session, small group intervention to reduce risky sexual behaviors and STDs among African American and Latina adolescent girls. This intervention is culturally and developmentally appropriate, and is delivered to groups of 2-10 participants. Through the use of group discussions, videotapes, games and exercises, the intervention addresses beliefs relevant to HIV/STD risk reduction, illustrates correct condom use, and depicts effective condom use negotiation. |
| 24 | Women's Co-op | Group | Women’s Co-Op is a woman-focused intervention that incorporates gender- and culture- specific skills training for crack-using African American women. The first 2 sessions are delivered to women individually, and focus on pre- and post-test counseling for HIV. The final 2 sessions are delivered to small groups of 2 to 5 women, and use a support-based format to help women develop skills that can reduce their risk of HIV. These sessions include the development of communication and problem solving skills that increase women’s sense of power and ability to cope with stress. |
| 25 | WHP | Group | The Women’s Health Promotion (WHP) intervention consists of four standard HIV education sessions (lasting about 6 to 9 hours) that address HIV transmission and prevention, sexually transmitted diseases, sexual and reproductive anatomy, condom practice, and condom negotiation skills. The eight additional sessions involve speakers on a variety of topics deemed relevant by participants, including general mental health, depression, cervical cancer, non-HIV-related partner communication, diabetes, nutrition, partner violence, oppression, and social justice. |
| 26 | EXPLORE | Individual | The EXPLORE intervention consists of 10 core counseling sessions delivered one-on-one to participants. The first sessions are intended to establish rapport between the counselor and the participant, and to provide personalized risk assessments. The remaining sessions cover topics such as sexual communication, knowledge of personal and others’ HIV serostatus when making sexual decisions, and the role of alcohol and drug use in risk behavior. |
| 27 | Hot, Healthy and Keepin' it UP! | Group | The Hot, Healthy, and Keeping it UP! intervention is a group-level counseling and skills training intervention for homosexual API men. The intervention, delivered to groups of approximately 8 men, consists of one 3-hour culturally tailored session with four key components: (1) development of positive self-identity and social support; (2) safer sex education; (3) promoting positive attitudes toward safer sex; and (4) negotiating safer sex. |
| 28 | Nia | Group | Nia is a video-based motivational skills-building small-group intervention consisting of 6- 10 participants in each group. The intervention includes videos, movie clips, and discussion to educate men about HIV/AIDS, elevate their mood, and entertain them while reinforcing information and motivating behavior change. Facilitators discuss with participants ways to prevent HIV/AIDS, including condom use, condom attitudes and the pros and cons of condom use, and teach problem-solving, safer sex, and decision-making skills. |
| 29 | SUMIT | Group | The Seropositive Urban Men’s Intervention Trial (SUMIT) enhanced peer-led intervention is delivered to groups of gay or bisexual men living with HIV in order to reduce risky sexual behavior. Led by HIV-seropositive gay or bisexual peer facilitators, structured group activities focus on sexual and romantic relationships, HIV and STD transmission, drug and alcohol use, assumptions about the HIV status of sex partners, disclosure of HIV status, and mental health. |
| 30 | BRAINE | Individual | BRAINE is a brief motivational interviewing intervention consisting of two individualized sessions focusing on alcohol use and HIV risk-taking. The first session, lasting 60 minutes, consists of assessing the participant’s degree of hazardous drinking, providing feedback, identifying relationships between drinking and negative consequences including HIV risk behaviors, reviewing HIV drug risk behaviors, and identifying personal goals and potential barriers for behavior change. |
| 31 | HIP | Group | The Health Improvement Project (HIP) intervention is a small group, skills training intervention to reduce risky sex behavior among persons with mental illness. Through interactive group discussions and motivational exercises, the first 4 sessions provide participants the facts about sexual behavior, HIV, and STDs; increase awareness of HIV risk. Through the use of role plays, the remaining 6 sessions provide participants the skills necessary to use male and female condoms, develop coping strategies to deal with risky situations, and negotiate condom use with sex partners. |
| 32 | Holistic Health Recovery Program | Group | Holistic Health Recovery Program (formerly Holistic Harm Reduction Program) is a 12-session, manual-guided, group-level program for HIV-positive and HIV negative injection drug users. The primary goals of HHRP+ are harm reduction, health promotion, and improved quality of life. |
| 33 | MIP | Individual | Modelo de Intervención Psicomédica (MIP) is an intensive intervention that combines counseling and case management. The 6 one-on-one counseling sessions conducted by a registered nurse use motivational interviewing strategies to engage injection drug users for behavior change. Participants also received standard HIV counseling and testing. |
| 34 | Safety Counts | Group | The Safety Counts intervention consists of a total of 9 sessions focusing on developing and implementing a personalized risk reduction plan. One month after the client receives the individual counseling session, a minimum of two 15-20 minute field-based supportive follow-up outreach contacts are scheduled to reinforce progress toward risk reduction and encourage achievement and maintenance of personal risk reduction goals. |
| 35 | SHIELD | Group | In the SHIELD model of HIV prevention, one individual (a Peer Educator) is taught strategies to reduce HIV risk associated with drug use and sex behavior. In addition, Peer Educators are taught effective communication skills in order to talk with people in their social networks about HIV prevention information. |
| 36 | Sniffer | Group | SNIFFER is a four-session, small-group, social learning based, AIDS/drug injection prevention intervention for intranasal drug users. The intervention is designed to create a support-group type of atmosphere so participants feel comfortable discussing personal problems and seeking help from the facilitators and their peers. |
| 37 | RAPP | Community | Real AIDS Prevention Project (RAPP) is a community-level intervention that mobilizes the networks of community volunteers, organizations, and business. The community contacts, activities, and materials provide tailored prevention messages and encourage behavior change to increase condom use among women. |

Appendix B. Survey instrument

Section A: Eligibility. Thank you for considering our survey. We have just a few questions to make sure that your organization meets our criteria and that you are the right person to answer the questions.

1.1 Does your organization provide services designed to prevent HIV, like individual counseling or group education programs?

- Yes
- No

1.2 Do you refer people interested in HIV prevention services to another location?

- Yes. Please list ________________________________________________
- No

1.3 Within the past ten years, has your agency implemented any of the following interventions for HIV prevention with people who are HIV negative? (please select all that apply)

- RESPECT or CRCS (Comprehensive Risk Counseling & Services)
- Some other type of counseling and testing program (please describe) ________________________________
- Doing Something Different
- "light"
- AIM (Adult Identity Mentoring)
- ARK (Assisting in Rehabilitating Kids)
- BART (Becoming a Responsible Teen)
- Be Proud! Be Responsible!
- ¡Cuídate!
- FOY (Focus on Youth)
- Focus on Youth + ImPACT
- SIHLE
- Street Smart
- Teen Health Project
- TLC (Together Learning Choices)
- (CE-AP) Communal Effectance-AIDS Prevention
- Choices
- FIO (Future is Ours)
- Insights
- SAFE
- Safer Sex
- Sepa
- SISTA
- Sisters Saving Sisters
- Women's Co-op
- WHP (Women's Health Promotion)
- EXPLORE (32)
- Hot, Healthy and Keepin' it UP!
- Nia
- SUMIT (Seropositive Urban Men's Trial)
- BRAINE (Brief Alcohol Intervention for Needle Exchangers)
- HIP (Health Improvement Project )
- Holistic Health Recovery Program
- MIP (Modelo de Intervención Psicomédica)
- Safety Counts
- SHIELD
- Sniffer
- RAPP (Real AIDS Prevention Project)
- Don't know/Not sure
- My organization has not implemented any of these interventions

1.4 In your position have you had oversight of the delivery of these kinds of interventions?

- Yes
- No

**Section B. Organizational and Participant Questions. This first set of questions ask about the background of your agency.**

2.1  How do you best describe your agency/organization?

- State Health Department
- Local Health Department
- Community-based Organization
- Health Care Facility (e.g., hospital, clinic, medical health center, or federally qualified health center)
- Other (please specify) ________________________________________________

2.2 Does your organization have collaborations (either formal or informal) with (please select all that apply)?

- A college or university
- Other health service agencies
- Social service agencies
- Faith-based organization
- For-profit business
- Local or state health department
- Other (please specify) ________________________________________________
- Don't know/Not sure

2.3 How many full time equivalent (FTE) positions are in your organization?______

2.4 For the last fiscal year, what was your total revenue amount (including all sources of financial support)?________________

2.5 Do you receive financial support from...? (please select all that apply)

- Federal grants or federally allocated money (e.g., HRSA or SAMHSA)
- State or local grants or allocated money
- Grants or donations from non-profit organizations (including foundations)
- Community/individual donations (including fundraising)
- Insurance biling
- Direct fees to clients for service
- Other (please specify) ________________________________________________

2.6 Please estimate the percentage of your clientele within each of the following categories.  (If you do not have any clientele that fall within a category, please put a zero.)

_______ % - 20 years or less

_______ % - 20-29 years

_______ % - 30-39 years

_______ % - 40-49 years

_______ % - 50-60 years

_______ % - 60 years or more

2.7 Please estimate the percentage of your clientele within each of the following categories.  (If you do not have any clientele that fall within a category, please put a zero.)

_______ % - Ethnic and/or racial minorities

_______ % - Men who have sex with men

_______ % - Injection drug users

_______ % - Transgender individuals

_______ % - Homeless

_______ % - Incarcerated or formerly incarcerated

_______ % - Low socio-economic status

_______ % - HIV positive individuals

2.8 My organization provides...

- HIV Testing
- STD Testing
- Both HIV and STD Testing
- Neither HIV or STD Testing

**The following questions are more about you, your background, and your position at your agency.**

2.9 Which of the following best describes your position?

- Program manager or Coordinator
- Program Evaluator
- Executive Director/Overall Director
- Division or Bureau Head/Deputy Director
- Department Head
- Other (please specify) ________________________________________________

2.10 How long have you worked for this agency? (In years)

*drop down selection

2.11 What is your highest level of education?

- High school or equivalent
- 2-year degree or Associates degree
- 4-year college degree
- Graduate degree
- Other (Please describe) ________________________________________________

2.12 How do describe yourself?

- A man
- A woman
- Gender nonconforming/Other

2.13 What is your age?

- 19 years or less
- 20-29 years
- 30-39 years
- 40-49 years
- 50-59 years
- 60-69 years
- 70 years or more

2.14 Do you consider yourself to be....?

- White
- Black or African American
- American Indian or Alaska Native
- Asian
- Native Hawaiian or Pacific Islander
- Other

2.15 Do you consider yourself to be Hispanic?

- Yes
- No

**Section C: The following questions will ask about the intervention(s) your agency has implemented.**

3.1  You will see each of the interventions you selected below.  Please fill in the date of initiation implementation, the date the program ended. If your organization continues to provide this intervention, check the box to the right. My organization…

|  | ... implemented this intervention on... | | ...ended this intervention on... | | My organization continues to provide this intervention. |
| --- | --- | --- | --- | --- | --- |
|  | (MM) | (YYYY) | (MM) | (YYYY) | Select Below |
| [*Each Intervention Inserted Here*] |  |  |  |  |  |

3.2  You will see each of the interventions you selected below.  Please check the appropriate boxes describing initial investments in the intervention.

|  | New staff were hired to provide or supervise this intervention. | | Staff were given specific training for this intervention. | | New materials or equipment were developed or purchased to provide the intervention (e.g., promotional materials, test kits, software). | | New physical space was used for the intervention (e.g., office space or clinic). | | New collaborations/ relationships outside my organizations to implement this intervention. | | New collaborations/ relationships within my organization to implement this intervention. | | External funding was used to start this intervention. | | Internal funding was used to start this intervention. | |
| --- | --- | --- | --- | --- | --- | --- | --- | --- | --- | --- | --- | --- | --- | --- | --- | --- |
|  | Yes | No | Yes | No | Yes | No | Yes | No | Yes | No | Yes | No | Yes | No | Yes | No |
| [*Each Intervention Inserted Here*] |  |  |  |  |  |  |  |  |  |  |  |  |  |  |  |  |

**Section D: The following section asks a set of questions about each of the intervention(s) your agency is continuing to offer.**

4.1 Of the initial funding and investments your organization made in [*intervention name*], how have these changed over time? Select N/A if your organization did not make an investment in this category.

|  | Increased | Stayed the same | Decreased | Totally Eliminated | N/A |
| --- | --- | --- | --- | --- | --- |
| External funding |  |  |  |  |  |
| Internal funding |  |  |  |  |  |
| Staff positions to provide intervention |  |  |  |  |  |
| Staff positions to supervise intervention |  |  |  |  |  |
| Physical space to provide intervention |  |  |  |  |  |
| Collaborations within the organization |  |  |  |  |  |
| Collaborations outside the organization |  |  |  |  |  |

4.2 What are some of the reasons your organization has continued [*intervention name*]? (Please select all that apply)

- Client Demand
- Staff Demand
- Fills a gap in agency services
- Funder support to implement
- A mandate of some kind
- Other (please specify) ________________________________________________

4.3 Of the reasons you mentioned, what is the primary reason your organization has continued [*intervention name*]?

- Client Demand
- Staff Demand
- Fills a gap in agency services
- Funder support to implement
- A mandate of some kind
- Other (please specify) ________________________________________________

**Section F: The following section asks a set of questions about each of the intervention(s) your agency discontinued.**

5.1 What are some of the reasons your organization has discontinued [*intervention name*]? (Please select all that apply)

- Loss of funding
- Not enough client demand
- Turnover or loss of staff with training in the intervention
- Lack of leadership support for the intervention
- Mandate of some kind
- Change in the mission or goals of my organization
- Insufficient evidence for its success
- Other (please describe) _______________________________________________

5.2 Of the reasons you selected, which is the primary reason your organization has discontinued [*intervention name*]?

- Loss of funding
- Not enough client demand
- Turnover or loss of staff with training in the intervention
- Lack of leadership support for the intervention
- Mandate of some kind
- Change in the mission or goals of my organization
- Insufficient evidence for its success
- Other (please describe)

5.3  Did your organization actively work to conclude [*intervention name*]? For example, did your organization notify clients or partners that the intervention was going to end?

- Yes
- No
- Not Sure

5.4 If yes, please describe the tasks staff engaged in to end [*intervention name*].

________________________________________________________________

________________________________________________________________

________________________________________________________________

________________________________________________________________

5.5 How would you rate the ease of ending [*intervention name*]?

- Very hard
- Somewhat hard
- Neither hard nor easy
- Somewhat easy
- Very easy

5.6 What, if any, aspects of [*intervention name*] continued after it was formally ended? (Please select all that apply)

- None of the aspects continued
- Retained newly hired staff
- Knowledge/skills learned by staff
- New physical space acquired
- New materials purchase
- New protocols developed
- Partnerships/Collaborations built with other departments inside the organization
- Partnerships/Collaborations built outside the organization
- Other (Please explain) ________________________________________________

5.7 Would you like to have continued offering [*intervention name*]?

- Yes
- Maybe
- No

5.8 Please tell us a little about why or why not.

________________________________________________________________

________________________________________________________________

________________________________________________________________

________________________________________________________________

5.9 Was [*intervention name*] replaced with another intervention?

- Yes
- No
- Not Sure

5.10 What was the name of the replacement intervention?

________________________________________________________________
